# Estimation of the Dissipative Heat Sources Related to the Total Energy Input of a CFRP Composite by Using the Second Amplitude Harmonic of the Thermal Signal

**DOI:** 10.3390/ma13122820

**Published:** 2020-06-23

**Authors:** Rosa De Finis, Davide Palumbo

**Affiliations:** Department of Mechanics, Mathematics and Management, Politecnico di Bari, 70125 Bari, Italy; davide.palumbo@poliba.it

**Keywords:** FRP composites, total energy input, thermography, second amplitude harmonic of thermal signal

## Abstract

Theories for predicting the fatigue behaviour of composite laminates often make strong assumptions on the damage mechanisms that strongly depend on the designed laminate lay-up. In this regard, several physical and empirical models were proposed in the literature that generally require experimental validations. The experimental techniques, such as thermography, also provide useful tools for monitoring the behaviour of the specific material so, that they can be used to support the study of the damage mechanisms of materials. In this research, the second amplitude harmonic of the thermal signal has been investigated and used to assess the relationship with the total energy input in order to estimate the fatigue strength of the material. A thermal index was assessed by monitoring the constant amplitude tests (S/N curve) that were performed on a quasi-isotropic carbon fibre reinforced polymer (CFRP) laminate obtained by the automated fibre placement process. The obtained results demonstrated the capability of the second amplitude harmonic of the thermal signal to describe and monitor the fatigue damage.

## 1. Introduction

The fatigue damage in a fibre-reinforced composite can be due to the presence of irregularities of the stress distribution into the material and the presence of mechanisms of damage accumulation that lead to the breakdown of the matrix or fibres or their interfaces [1,2,3,4].

In general, the composite behaviour during cyclic loading involves three stages of the damage: an initial decrease of the mechanical properties due to the matrix cracking, a stable mechanical degradation growth due to the occurrence of delamination induced by transverse cracks, and a final severe degradation due to the fibres rupture [4,5,6,7,8,9]. These three stages of damage can be assessed by evaluating the material performances in terms of the hysteresis loop changes (rotation and drift) [1,10]. To this purpose, methods that are based on the assessment of Young’s modulus [7,8,9] and Poisson’s ratio [11] reduction, crack density [12,13,14], and strain density variation [2,15] measurements can be used for describing the material behaviour. The stiffness degradation in terms of Young’s modulus, Poisson’s ratio reductions, and crack density evaluations are useful for describing the material response [2] and are representative of specific mechanisms, such as transverse cracks, shear stresses at the interfaces, and the appearance of the characteristic damage state [16]. In this regard, the assessment of the evolution of the area of the hysteresis loop is more promising for describing the material behaviour, as it represents the total energy input (strain energy density) [2].

Several methods were developed to obtain the total energy input [2,10,17,18,19] in order to draw considerations on material residual strength. By considering that the imposed stress is the main damage controlling parameter [1] under a fatigue regime, Ellyn [2] proposed a model that related the total energy input to stress/strains in the material principal directions, for elastic plane stress problems of orthotropic laminates. Plumtree et al. [20] proposed another formulation, where the total energy input that was related to in-plane normal and shear stresses and strains. Varvani-Farhani [19] proposed a model that was based on the physics and the mechanisms of cracking within the three stages of damage for CFRP composites. In the model, the energy-based fatigue damage parameter was the sum of the three contributions to the total energy input in the three typical damage stages [4]. However, the approach requires calculation and assumptions that are based on classical lamination theory (CLT) at any given stress cycle and the assessment of static material properties.

The presented methods consider the energetic state of the material at a specific stress/strain level, but they do not allow for the assessment of the evolution of the energy through the cycles. Moreover, they also require specific assumptions on the damage phenomena and their interactions. However, they can be considered as a reference for the estimation of total energy input.

The total energy input can be also evaluated by means of experimental approaches. In effect, several experimental techniques [21,22,23,24,25,26,27,28,29,30,31,32,33,34,35,36,37,38] were used in the last decades capable of assessing the evolution of the total energy input and studying the damage. Behera [21] used optical microscopy coupled with the measurements of the load-displacement area under the hysteresis loop that was provided by an automatic data acquisition system to study stiffness degradation and material energy dissipations. The approach is useful for structural health monitoring assessment, but, to perform quantitative microscopy analysis, the sample has to be removed from the loading machine grips. In view of this, Liu [22] tested a T300/924 laminated composite at various porosity levels, and showed the effects of voids on fatigue behaviour (stiffness variations, initiation/propagation of matrix cracks, and fatigue failure mechanisms).

Acoustic emissions (AE) were used in several works [23,24,25] for determining the time instant at which the damage occurs and for characterising the fatigue damage. However, AE present some limit in localizing the damaged areas and the heat sources due to the overlapping of acoustic signals from different damage sources. The strain gages (SG) [26] allow for the strain analysis of small regions of interest of the component, while the Digital Imagine Correlation [27,28] is a full-field technique that provides the superficial strain maps of the component. These latter techniques are very useful for obtaining the strain energy input, but they require specific surface preparation for applying the sensors (SG) and for applying the speckle pattern (DIC).

Toubal [29] used the infrared thermography technique to study the damage evolution of woven laminates by measuring the material surface temperature during fatigue tests. This approach has the advantage of monitoring the material state in real-time during the fatigue tests. In the work of Montesano [30], a direct relation between the temperature and the energy dissipated evaluated as the area under the hysteresis loop in a loading cycle is presented. This approach led to obtaining the S/N curve. Huang et al. [4] proposed a model that was capable of reconstructing S/N curve based on the stiffness degradation and temperature measurements acquired during rapid fatigue tests. However, the temperature is a physical quantity that can be influenced by external noise factors: heat exchanges by convention with the external environment or the thermal conduction through the grips or fixtures [31]. Another application of thermography is provided by Sarker [32]. In the work, Sarker showed as the active stepped thermographic technique coupled with EDX measurements applied on a CFRP and epoxy samples after cyclic loading can be a useful tool for studying the effect of different sulphuric acid environments on the thermo-physical and mechanical properties of these materials and their constituents.

In another approach [33], the maps of the thermoelastic behaviour of the material during fatigue loading presented different signal changes that were related to the stiffness variations and stress redistribution that can be used to study the material degradation. In effect, Shiozawa [34] presented that the damage evolution can also be assessed using thermoelastic signal and phase shift, on Short Carbon Fiber Reinforced Plastics. In particular, in that work, the evolution of the damage was obtained by a new thermoelastic phase damage analysis that improves the phase contrast for detecting damage on these specific materials.

In recent years, the thermal signal analysis approach was developed for assessing the different harmonics components of the thermal signal that are related to both thermoelastic and dissipative heat sources [35]. Following this approach, Palumbo et al. [36] showed a new method to filter out ‘disturbing’ heat sources affecting temperature signal based on the analysis of spectral components of the temperature. In particular, in that work, it was showed in a qualitative way that the amplitude of the second harmonics of the thermal signal of a GFRP laminate undergoing cyclic loading was representative of the intrinsic dissipations and it can then be used to detect and monitor the damage. In effect, thermal signal components can describe local temperature variations related to the damage in the material [35]. This is particularly important for an accurate detection of the damage locations. More recently, De Finis et al. [37] presented a preliminary qualitative study of the relation between the first harmonic amplitude (thermoelastic temperature signal) and stiffness variation evolutions in a quasi-isotropic CFRP for this material, in the work the capability of thermoelastic stress analysis to predict the failure area was also presented.

In this work, by using the same material and experimental set-up in [37], another different physical thermal index has been used for studying the fatigue behaviour of CFRP composites. The second amplitude harmonic of the thermal signal has been used for correlating the total energy input and dissipative heat sources. The main novelty of the present research consists in the experimental investigation of the relationship between the second amplitude harmonic of the thermal signal and the total energy input obtained by evaluating the area under the hysteresis loop with an extensometer during the same tests. Moreover, in the present manuscript, by using the Ellyn model [2], the stresses of the S/N curve have been obtained via energy input using mechanical and thermal data.

The advantage of the present approach is the possibility of realising real-time monitoring of the damage of real components where it is difficult to measure the total input energy with traditional approaches.

## 2. Theory: Fatigue Energy Assessments in Composites

### 2.1. Total Energy Input

Under cyclic loading, the mean and maximum values of the applied stress levels control the initial and final damage stages [1]. In these conditions, damage and material properties degradation occur in a cumulative way [19]. In effect, the major part of the laminate life is dominated by a subcritical damage accumulation that manifests in the form of matrix/fibres cracking, delamination, debonding, etc. [2].

According to different authors [2,3,19], the fatigue life of the material is related to the total energy input per unit volume per cycle by:

(1) $$W=A\;N^{a},$$

where *A* and *a* are constants depending on loading ratio, fibre orientation, while *N* represents the cycles run. The same power law is used for describing the relation between stress and cycles [15], which is the S/N curve.

The formulation requires defining a proper constitutive law describing the material response in order to theoretically describe the total energy input [2,15,19,20].

For an elastic plane stress problem, Ellyn [2] expressed the total energy input (strain energy density) in terms of the stress and strains and loading ratio in the case of uniaxial cyclic loading:

(2) $$W=\frac{{\bar{S}}_{11}}{2{\left(1-R\right)}^{2}}\Delta \sigma^{2},$$

where Δσ is the stress amplitude, *R* is the loading ratio ($\sigma_{min}/\sigma_{max}$) and ${\bar{S}}_{11}$ can be described by:

(3) $${\bar{S}}_{11}=\frac{1}{E1}\cos^{4}\theta +\frac{1}{E2}\sin^{4}\theta +\left(-\frac{v}{E1}+G_{12}\right)\sin^{2}\theta \cos^{2}\theta ,$$

where ${\bar{S}}_{11}$ is the transformed compliance value that depends on the angular orientation of the fibres with respect to the load direction *θ* and on mechanical properties, such as elastic moduli in longitudinal, transverse directions (*E*_1_, *E*_2_), shear modulus (*G*_12_), and Poisson’s ratio *v*_12_. The material properties can be described by the constants *E* and *G* for an elastic isotropic laminate under uniaxial loading conditions *θ* = 0 (the principal material directions and loads are in the same system reference). It leads the transformed compliance is simply described by ${\bar{S}}_{11}$ = 1/E.

The model provided by Equation (2) is taken into account for the fatigue strength estimations starting from the total energy input measurements.

### 2.2. Second Amplitude Harmonic of the Thermal Signal

Under cyclic loading, the mechanical energy (area under the hysteresis loop) is transferred into the material [10]. This energy converts both into heating and for the damage production and propagation [15,19,35]; hence, it is responsible for the material response. The characterisation of actual damage state of the material can be assessed by measuring the portion of the total energy that converts in heating as about the 80–85% of the total energy [10].

In the absence of heat exchanges (*Q*) (adiabatic conditions), the total energy input per unit volume in a cycle) (*W*) can be directly related to the internal energy term (ΔU) represented by the energy stored in the material (*E_s_*) and the energy dissipated as heat (*E_d_)* [38,39]:

(4) $$W=\oint \sigma d\varepsilon =Q+\Delta U=Q+E_{s}+E_{d},$$

where *σ* and *ε* are respectively the stress and the strain of the hysteresis loop. For certain types of composites, due to the linear elastic behaviour, it is possible to assume that the major portion of the supplied mechanical energy converts into heat [10].

The temperature can be considered a feasible estimator of the energy involved in the dissipative processes, as demonstrated by several researchers [31,35,36]. However, the measurement of the temperature of the material during fatigue tests depends on several noise factors (environment heat exchanges, conductive heat exchanges with grips) that can blur the signal of the damage heat sources. A suitable smooth of the data is required for filtering out these external factors and improving the signal to noise ratio.

Several authors [35,40,41], adopted a suitable approach to decompose the mean temperature variations from the sinusoidal ones This approach leads to a more accurate description of the material state by evaluating different temperature effects that are related to the energy heat sources. The thermal signal can be represented by mean temperature variations (the well-established parameter used by [31]) and components running at the mechanical frequency and at the twice the mechanical frequency, which are representative of the thermoelastic [36] and dissipative phenomena [42], respectively. The thermoelastic temperature component describes phenomena, such as stiffness degradation and stress redistribution [43], while the second amplitude harmonic, as demonstrated by Enke [42], can be used to estimate the energy involved in damage processes.

In a previous work of Palumbo et al. [36], the second amplitude harmonic of the thermal signal has been used for evaluating the fatigue strength with a rapid procedure. In particular, it has been demonstrated, as the second-order harmonic provides results that are in agreement with the traditional approaches and allows for localizing and identifying the damaged areas with better accuracy than the traditional approaches [35]. In the present work, the second amplitude harmonic of the thermal signal will be used for estimating the heat dissipated during the fatigue damage and it will then be related to total energy input for evaluating the S/N curve of the material.

## 3. Material and Experimental Campaign

**Figure 1:**
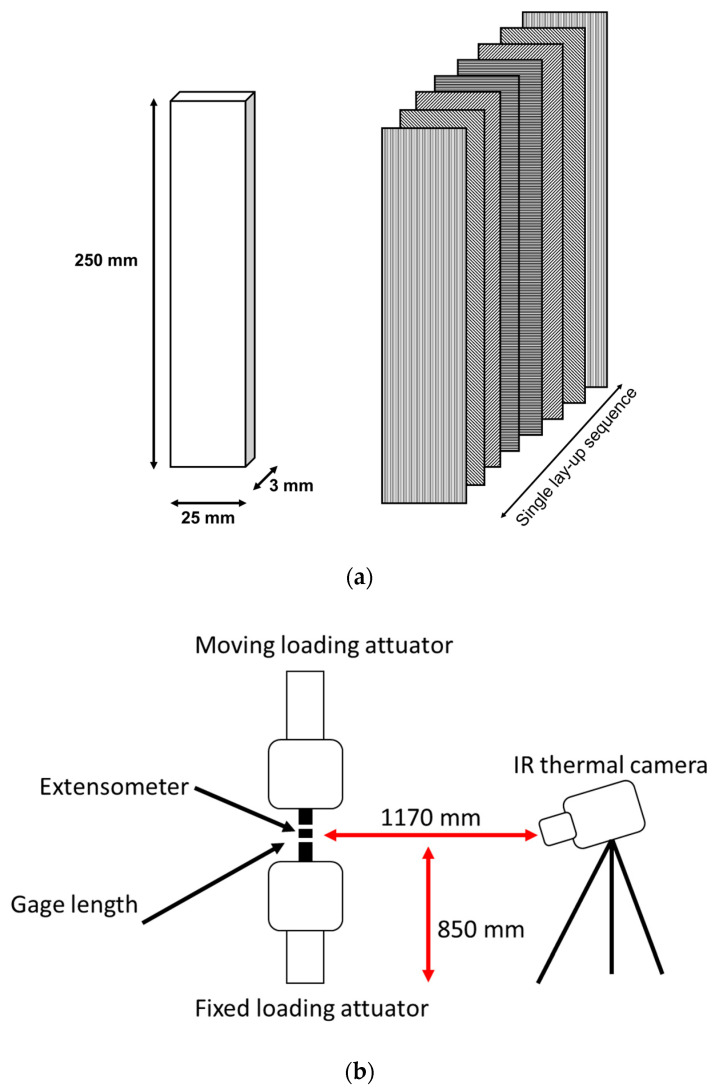
(**a**) sample geometry and dimensions, (**b**) setup and equipment.

The tested material is a quasi-isotropic CFRP made by Automated Fiber Placement (AFP) [44,45], an innovative process for the automated deposition of the dry-fibres/thermosets/thermoplastics plies. The roller of the robotic arm has a longitudinal dimension of 60 mm and a radial dimension of 69 mm. The process deposition speed varies between 320 and 480 mm/sec [39,40]. The samples were obtained from a panel with fifteen plies of an epoxy-type resin reinforced by carbon fibers, each ply has roughly the same thickness of 0.183 mm. The lay-up sequence [0/−45/45/90/90/45/−45/0]_2_. The sample geometry, as in Figure 1a, involves a square cross-section and a constant gage length. The sample width is 25 mm, the length is 250 mm, and the thickness is 3.0 mm. Tensile static tests that were performed on five samples at the displacement rate of 1 mm/min. provided the average value of the Ultimate Tensile Strength (UTS) and Young’s modulus, the results are reported in Table 1.

Constant amplitude fatigue tests have been performed on 10 samples (one sample tested at each stress level) at the stress ratio of 0.1, loading frequency of 7 Hz, under load control. The cycles runout was 2 × 10^6^ cycles.

Table 2 reports a resume of the imposed stress levels and cycles run to build the S/N curve.

Table 2. also reports the percent of Ultimate Tensile strength (%UTS) to which maximum stresses are referred.

The fatigue tests have been carried out by using a 25 mm gauge length contact clip-on extensometer for measuring the strain. The frame rate used for acquiring mechanical data was 100 Hz.

Thermal sequences were acquired by a cooled In-Sb detector FLIR X6540 SC (640 × 512 pixel matrix array, thermal sensitivity NETD < 30 mK) with a frame rate of 177 Hz. Each thermal sequence acquisition is composed by 1770 frames acquired. The experimental set-up allowed for obtaining a millimetre-to-pixel ratio of 0.35.

Figure 1b shows the setup and equipment. The infrared camera was positioned in front of the monitored surface of the sample.

## 4. Data Processing

### 4.1. Hysteresis Loop Measurements (W)

The data obtained by the extensometer have been processed to assess the area under the hysteresis loop to obtain the total energy input *W* per cycle. The values of *W*, assessed for each recorded cycle, allows for the monitoring of the energy during the fatigue life for a fixed value of the stress (UTS%).

**Figure 2:**
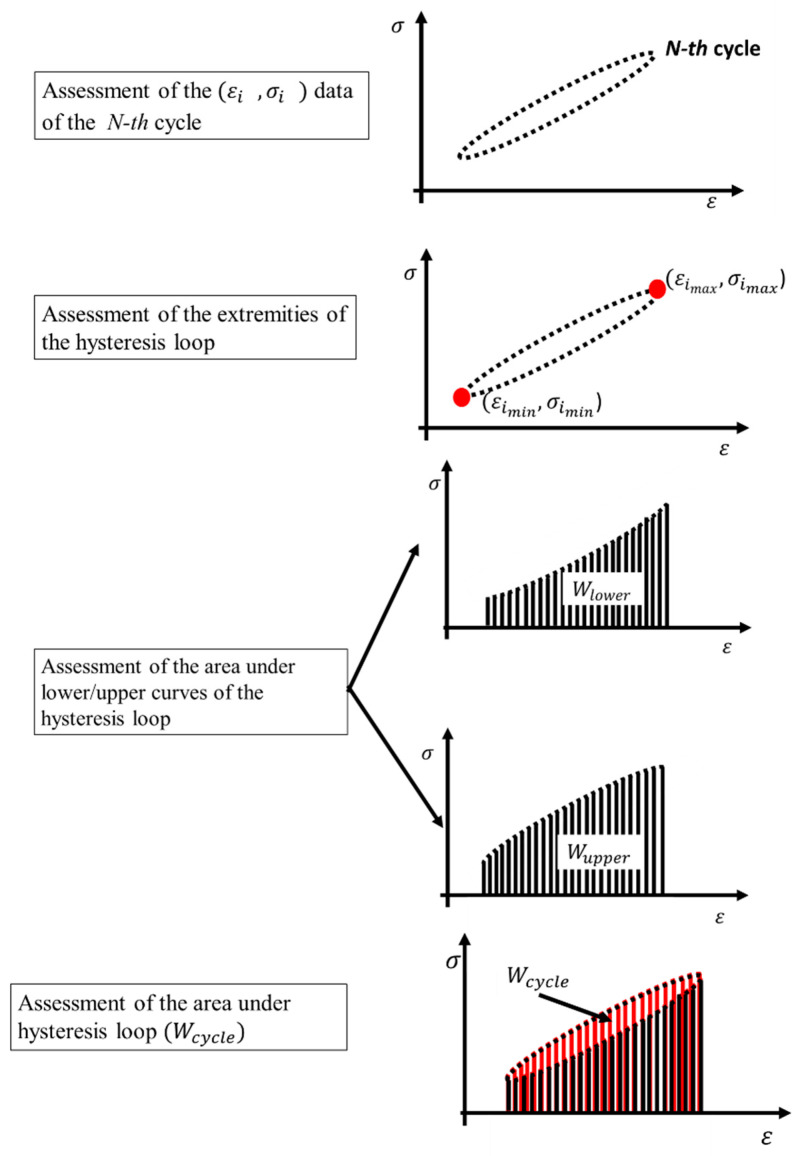
Procedure for assessing the area under the hysteresis loop.

The procedure of analysis (Figure 2) of the extensometer data implemented in Matlab^®^ (Mathwork, Natick, MA, US) involves the following steps:

- assessment of the strain-stress couples (*ε_i_*, *σ_i_*) of the *N*-th cycle. One cycle is composed of 16 (*ε_i_*, *σ_i_*) data couples;
- finding the maximum (minimum) stresses (*σ_max_*) *_imax_* and (*σ_min_*) *_imin_* where the indexes *imax* (*min*) represent the values of the index *i* in correspondence of the maximum (minimum) stresses, respectively, *σ_max_* and *σ_min_*;
- finding the extremities of the hysteresis loop in term of the couples:
  ○ ε(σ_max_)_imax_, (σ_max_)_imax_;
  ○ ε(σ_min_)_imin_, (σ_min_)_imin_;

clearly, the values of the strains were evaluated in correspondence of the maximum (minimum) stresses.

- integration of the stress-strain data (*ε_i_*, *σ_i_*), *i = i_max_*:*i_max_* + 8, via the trapezoidal method to assess *W_lower_*;
- integration of the stress-strain data (*ε_i_*, *σ_i_*), *i = i_min_*:*i_min_* + 8, via the trapezoidal method to assess *W_upper_*; and,
- assessment of energy input per cycle *W = W_upper_* − *W_lower_*.

The presented procedure allows for obtaining the evolution of the energy input through the loading cycles.

### 4.2. Thermal Signal Analysis

The thermal signal was analysed by means of the IRTA^®^ software [46] that allows for processing thermal data by using the following model (least squares method):

(5) $$S\left(t\right)=S_{0}+S_{1}\sin\left(\omega t+\varphi_{1}\right)+S_{2}\sin\left(2\omega t+\varphi_{2}\right),$$

where *S*_0_ is the mean temperature signal, while *S*_1_, *S*_2_ are the amplitude of the first and the second harmonics and *φ*_1_, *φ*_2_ the related phase shifts. The angular frequency *ω* is proportional to the mechanical frequency. The output of the analysis are the maps where each pixel represents the value of the thermal signal components [47].

In the present research, the component taken into account is *S*_2_. Subsequently, the *S*_2_ data were processed to reduce the noise and extract the thermal index (thermal metric) related to *S*_2_ from each image.

**Figure 3:**
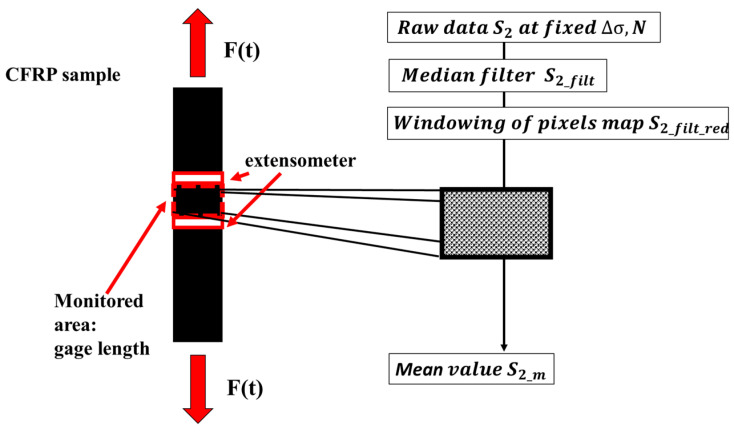
Procedure for processing the second harmonic amplitude of the thermal signal.

The algorithm for the data processing has been implemented in a suitable Matlab^®^ routine, whose basic steps are graphically described in Figure 3.

The processing involves the:

- application of the two-dimension spatial median filter (Medfilt2) to obtain *S*_2*_filt*_. Each output pixel contains the median value in a 3-by-3 neighbourhood around the corresponding pixel in the input image;
- reduction of the thermal scene to the gage length area to obtain *S*_2*_filt_red*_, to make the signal value in the same area considered for extensometer analysis; and,
- evaluation of the mean value of the signal *S*_2*_m*_.

## 5. Results

In this section, the evolution of *W* and *S*_2*_m*_ is presented and the proper value of each metrics representing the damage state at the specific stress level is also assessed.

### 5.1. Area under Hysteresis Loop (W) and Evolution through Constant Amplitude Loadings

**Figure 4:**
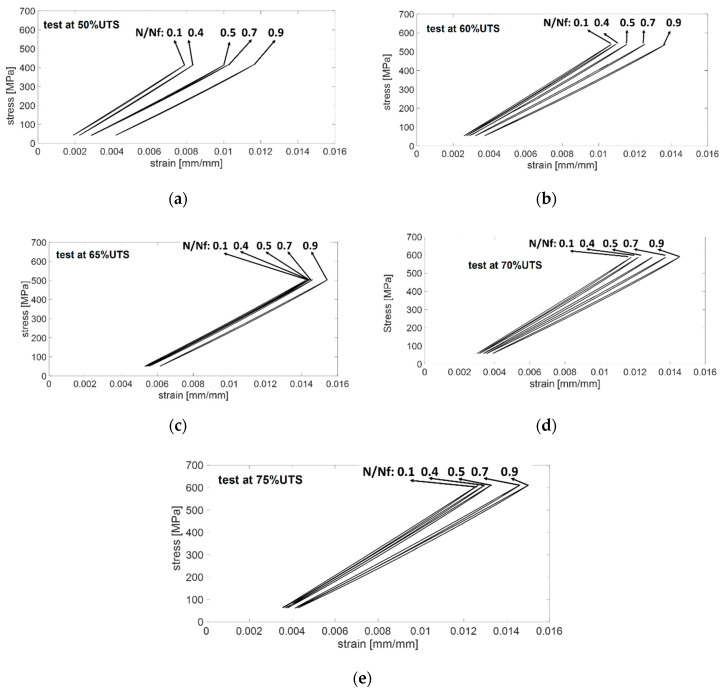
Hysteresis loops at (**a**) 50% UTS, (**b**) 60% UTS, (**c**) 65% UTS, (**d**) 70% UTS, and (**e**) 75% UTS.

For all of the stress levels the evolution of the hysteresis loop at specific cycles, in terms of *N*/*N_f_*, are presented in Figure 4.

The hysteresis ellipse-shaped loop is due to the viscoelastic characteristics of the polymer matrix and the friction between debonded and delaminated surfaces [39,48]. For the test at 50% UTS, Figure 4a shows narrow curves from the initial to the end of the test (2,000,000 cycles). The curves underwent a significant shift at higher strain values at roughly 50% of the lifespan (1,000,000 cycles) that continues up to the test stop. A curve rotation is also present, starting from 50% of the life span of the sample. The same behaviour (translation and rotation) of the hysteresis loops is observed in Figure 4b (test at 60%UTS) and Figure 4d (test at 70%UTS) and Figure 4e (test at 75%UTS), while the rotation is less marked in Figure 4c. In these cases, the area under the hysteresis loop is wider than the one at 50% UTS. The area under the hysteresis loop depends on the damage, as the imposed stress increases the damage increases, the curves are hence wider. These behaviours indicate a ratcheting deformation and a dynamic stiffness reduction, as observed in the literature [48].

The area under the hysteresis loop per cycle has been evaluated according to the procedure that is presented in Section 4.1.

**Figure 5:**
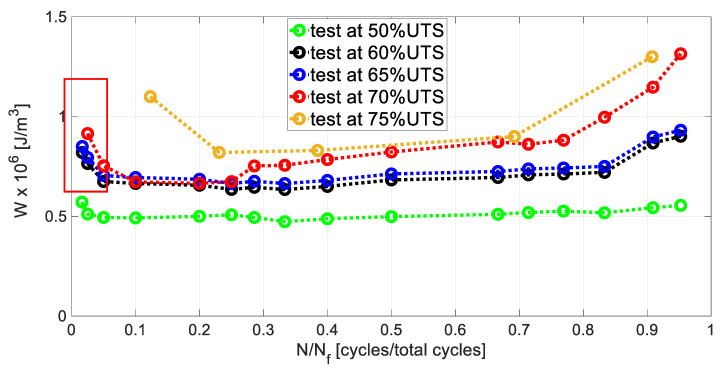
Evolution of *W* through *N*/*N_f_* for each stress level.

For each stress level, the *W* evolution per cycle is reported in Figure 5 as a function of *N*/*N_f_* that is the ratio between cycles and cycles at failure. The failure is represented by. In an early period of the evolution of *W*, it seems that the stiffness controls the energy, in fact, due to the appearance of transverse cracks, stiffness undergoes a copious reduction [4,39]. The initial reduction is due, in this case, to an initial degradation of the stiffness of the material that leads to an energy decrease for all the curves in Figure 5. Subsequently, a stabilization of the stiffness of the material, due to stable growth of the damage, also causes a stabilization in the energy per cycle. The final increase in the energy *W* is due to the damage increase through the simultaneous appearance of irreversible processes near the material failure (test at 60-65-70-75% UTS), while, for the loading level where the material did not fail (test at 50% UTS), this increase is not observed. In addition, the slope of the final energy increase clearly depends on the stress imposed, in fact, it is more severe for the tests that were carried out at 70–75% UTS, since more severe is the damage intensity at this stress level. In effect, at a higher stress level, the different damage processes occurring simultaneously lead to an abrupt increase of the total energy *W* [10].

The stress dependence is clearly visible in the *W* curves of Figure 5 in terms of the offset between the curves of each test. In particular, it is significant between the test at 50% UTS and the tests at 70–75% UTS, specifically at higher *N*/*N_f_*. This can be explained by the different damage mechanisms activated at lower and higher stress levels imposed. On the other hand, the initial data scatter between the curves can be due to the different amount of matrix driven processes (such as transverse cracks [4]) that depend on the stress level [16]. Moreover, *W* depends on material characteristics and the evolution depends not only on the stacking sequence, but also on the presence of flaws [2].

### 5.2. Second Harmonic Amplitude of the Temperature Signal Related to the Energy Dissipated

In this section, the maps of *S*_2*_fil_red*_ and the evolution of *S*_2*_m*_ processed according to the algorithm of the Section 4.2 are presented.

**Figure 6:**
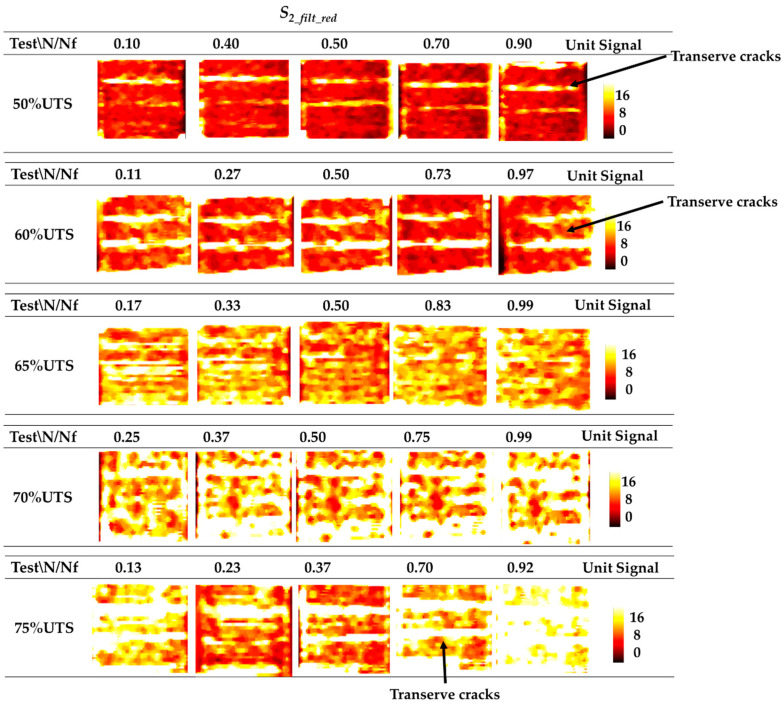
Pixels maps of *S*_2*_fil_red*_ signal at 50% UTS, 60% UTS, 65% UTS, 70% UTS, and 75% UTS at different *N/N_f_*.

In Figure 6, the maps of the second harmonic amplitude of the temperature signal are reported for the tests at 50% UTS, 60% UTS, 65% UTS, 70% UTS, and 75% UTS for fixed *N*/*N_f_* ratios referring to the gage length of the sample (between the blades of the extensometer).

The maps of Figure 6 show that the signal *S_2_fil_red_* of the test at 50% UTS exhibits a slight increase in the maximum and mean values through the cycles for all of the tests. The *S_2_fil_red_* of 60–65% UTS presents higher signal values than the previous test, a constant mean value between *N/N_f_* ≅ 0.3 and *N/N_f_* ≅ 0.73 and a small signal increase at *N/N_f_* ≅ 1. For the test at 70% UTS, the signal is slightly increased from *N/N_f_* = 0.37 to *N/N_f_* = 0.99. At 75% UTS, a decrease in the signal map *S*_2*_fil_red*_ up to *N/N_f_ =* 0.37 and the final increase at *N/N_f_ =* 0.7–0.92 are evident.

The *S*_2*_fil_red*_ signal increase in Figure 6 is appreciable from lower (first row) to higher (latter row) imposed stresses according to the *W* energy increase among stress levels. Moreover, in Figure 6, for each tested stress level, it is possible to see the transverse cracks [7,8,9]. The presence of transverse cracks affects the mechanical behaviour of the material in terms of stiffness reduction. In effect, the material stiffness reduces as the number of transverse cracks per unit length increases (crack density [12,13,14]). Clearly, this produces a variation in the energy, *W*.

**Figure 7:**
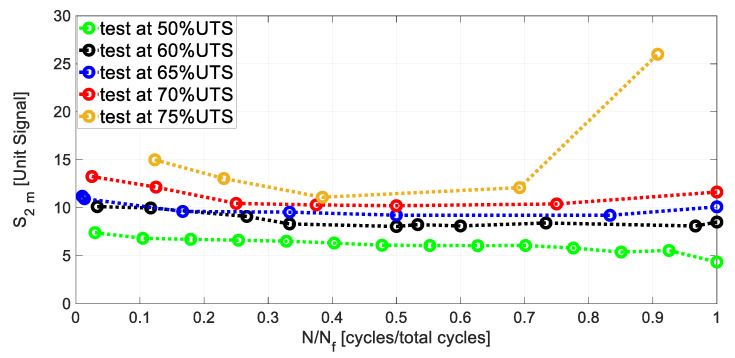
Evolution of the *S*_2*_m*_ through *N/N_f_* for each stress level.

Figure 7 represents the *S*_2*_m*_ through *N/N_f_*. The signal in the figure, for each stress level, presents an initial decrease and a stable phase. The slope of the *S*_2*_m*_ signal decrease is not very pronounced for all of the performed tests. A small final signal increase is observed in the tests at 50-60-65-70% UTS, while *S*_2*_m*_ increases abruptly for the test at 75%UTS. As previously said, *S*_2*_m*_ represents a parameter that is capable of estimating the portion of the energy input, *W*, which converts into heating. The small signal increase *S*_2*_m*_ in the last part of the test is in accordance with the behaviour of *W.*

It is worth noting to highlight that the *S*_2*_m*_ represents an estimator of the heating processes appearing on the material surface, so that it represents an index of the portion of the total amount of the heat dissipated in the material. This could justify the less pronounced decrease/increase in the trend of the curves of Figure 7. Referring to the curves of Figure 7, a small data offset is observed between the curves.

## 6. Discussion

### 6.1. Procedure to Assess a Damage State Parameter for the Specific Stress Level

This section introduces a procedure for evaluating an estimator of the material damage state that is understood in the present research as the characteristic value of *W* and *S*_2*_m*_ representative of the damage level at the specific imposed stress level. It is used to estimate the fatigue strength of *S/N* curve according to the procedure that will be further explained.

The metrics *W* and *S*_2*_m*_, as previously explained, have two different physical meanings; however, the *S*_2*_m*_ can be used to estimate the portion of *W* that converts into heating. In view of this, clearly the behaviours are similar, in effect the curves of both *W* and *S*_2*_m*_ present an initial decrease a steady state and a final increase of the signal. By taking the steady state of each parameter that occurs mostly in the range of 0.2–0.5 *N/Nf* for each stress level into account, it is possible to extract two parameters for assessing the damage level. In this indicated period of the sample-life, quasi-constants values are observed in the curves of Figure 5 and Figure 7 of *W* and *S*_2*_m*_, respectively. Accordingly, the mean values of these latter (*W_mech_*, *S*_2*_M*_) have been considered as representing the damage state of the material for each stress level:

(6) $$W{\;}_{mech}=\frac{1}{S}\sum_{i=1}^{S}W_{i},$$

(7) $$S{\;}_{2\_M}=\frac{1}{S}\sum_{i=1}^{S}S_{2\_m_{i}},$$

where *i* is the index that corresponds to the data points (*N/Nf _i_*, *W_i_*) and (*N/Nf _i_*, *S*_2*_m i*_) between *N/Nf* = 0.2 and *N/Nf* = 0.5 and *S* corresponds to the total number of data points in this range. *S* varies among the tests, but, in general, its value is at least three. For example, between *N/Nf* = 0.2 and *N/Nf =* 0.5, for the sample tested at 50% UTS, there are four data points, the *W_mech_* and *S*_2*_M*_ will be the averaged values of four data points lying in the considered interval.

Table 3 resumes the values that were calculated for each parameter.

**Figure 8:**
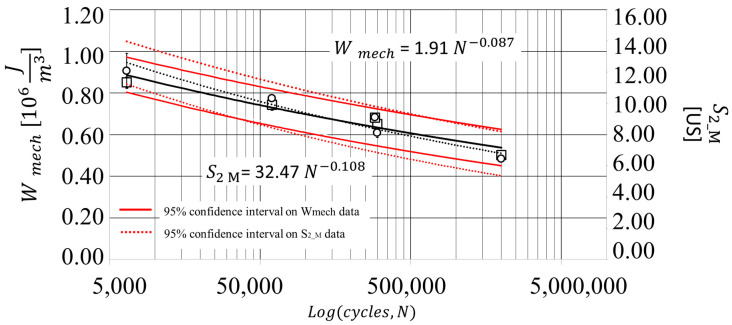
Comparison between *W_mech_* and *S*_2*_M*_ versus loading cycles *N*, with standard error bars and confidence levels.

In Figure 8, the total energy input (square marker) and the thermal parameter (round marker) are compared to cycles run *N*. It is possible to observe that a power law describes the behaviour of *S*_2*_M*_ as a function of the loading cycles. The exponent of the power fitting of the (*N*, *S*_2*_M*_) data is similar to the one of the (*N*, *W_mech_*); moreover, the two behaviours are similar. Figure 8 also reports the 95% confidence intervals that are represented by red dotted lines for *S*_2*_M*_ and red solid lines for *W_mech_*, with a minimal difference between the slopes. The error bars are also reported in Figure 8, representing the one-sigma (68% confidence interval).

**Figure 9:**
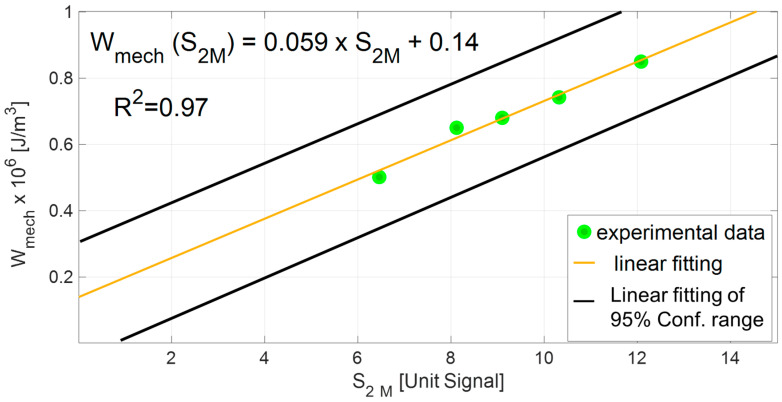
Relation between *W_mech_* and *S*_2*_M*_.

The relation between the total energy input and the thermal parameter can be finally investigated by plotting in the same graphs the *W_mech_* as a function of and *S*_2*_M*_, as in Figure 9. The linear regression shows the good correlation between the second harmonic amplitude of the thermal signal and the total energy input, as also demonstrated by the *R*^2^ coefficient of 0.97. Figure 9 also reports the linear regression lines of the 95% confidence interval that is presented in Figure 8, of *W_mech_* plotted as a function of *S*_2*_M*_.

The equation of the linear regression model in Figure 9 can be rewritten as:

(8) $$W_{mech}=W_{mech}\left(S_{2\_M}\right)=KS_{2\_M}+k,$$

where *K*, *k*, are the calibration constants of linear regression models, also reported in Figure 9 (*K* = 0.059, *k* = 0.14). The value of the total energy input *W_mech_* (*S*_2*_M*_) obtained through the model of Equation (8) can be recalled as *W_therm_*. In other words, the presented analysis demonstrated it is possible to obtain values of total energy inputs by simply analysing the temperature signal component.

In the following section, the data of *W_mech_*, *W_therm_* are used to assess the fatigue curve of the material.

### 6.2. Assessment of the Fatigue Curve by Means of Total Energy Input Estimations

In the present section, the fatigue stress assessments are presented by using the model that was proposed by Ellyn [2] (Equation (2)), opportunely rewritten as a function of the maximum stress σ:

(9) $$\sigma =\sqrt{W\;(2\;E\;\left(1-R{)}^{2}\right){\left(\frac{2}{1-R}\right)}^{2}},$$

The model requires as input the quantity R that is 0.1. The ${\bar{S}}_{11}$ of Equation (2), for an elastic isotropic laminate, coincides to the inverse of the measured Young’s modulus (E), which, in this case, is 55,800 MPa.

The input data W are represented by the values of the total energy inputs *W_mech_* and *W_therm_* and the output data are represented by the maximum stresses σ evaluated for each one of the total input series, respectively, *σ_Wmech_*, *σ_Wtherm_*.

Table 4 reports the assessed values of the maximum stress obtained by using, as input, the different contribution to total energy input *W_mech_*, *W_therm_*. By observing the values of *σ_Wmech_*, *σ_W therm_*, of Table 4, it arises that improvements in the precision and accuracy will be surely achieved by performing more tests and using another suitable model to correlate *W_mech_*, *W_therm_* and stresses of the S/N, but the discussion of the analytical model relating these quantities is not in the purpose of the present research.

**Figure 10:**
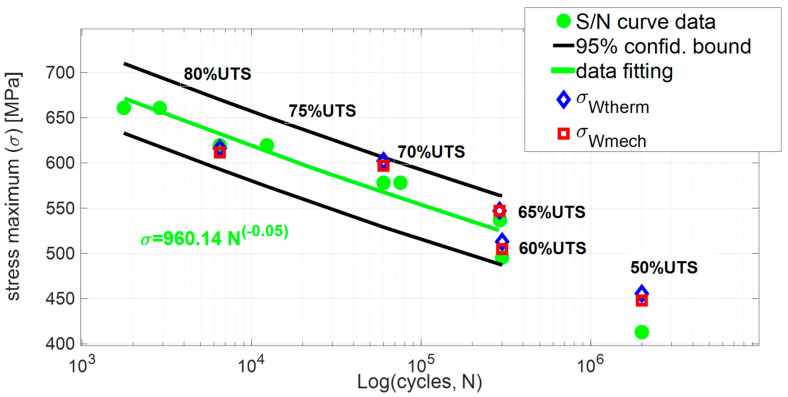
Stresses assessed *σ_Wmech_* (red marker), *σ_Wtherm_* (blue marker) by using the *W_mech_*, *W_therm_* data compared to S/N imposed stresses (green marker).

Figure 10 presents the stress assessments *σ_Wmech_*, *σ_Wtherm_* as compared to the imposed stresses of the S/N curve, respectively, represented by red, blue, and green markers.

As for the *σ_Wmech_*, the stresses values of the tests at 75% UTS are in good agreement with the stress of the S/N curve. All of the obtained stresses *σ_Wmech_*, *σ_Wtherm_* lie in the 95% confidence interval.

The stress values obtained by the second amplitude harmonic of the temperature signal, *σ_Wtherm_*, matches very well the imposed stress data, blue markers in Figure 10. Moreover, it is worth noting that the exponent of the power law describing the data (*N*, *S*_2*_M*_) in Figure 8 is very close to the one of the S/N curve of Figure 10 demonstrating the capability of *S*_2*_M*_ to reproduce the material strength.

Together with the advantages of the present approach, to perform the tests with a contactless technique, the main limitations refer to the possibility of accessing into the monitored areas with the IR camera and, in the presence of thick components, the difficulties to have a significant thermal signal related to the inner damage.

## 7. Conclusions

In the present research, the fatigue behaviour of quasi-isotropic CFRP samples that were obtained by automated fibre placement has been investigated using thermography. The aim of the research was to investigate the relationship between the total energy input and the second order amplitude harmonic of the thermal signal.

Specifically, the extensometer has been used for the assessment of the area under hysteresis loop that represents the total energy input, while the infrared detector has been used for the evaluation of the amplitude of the second harmonic of the thermal signal.

The first outcome refers to the estimation of a damage state parameter for total energy input and second amplitude harmonic (*W_mech_*, *S*_2*_M*_*)* representing the damage level at the specific imposed stress level. It was demonstrated that *W_mech_*, *S*_2*_M*_ exhibited a linear relationship with a good correlation. Moreover, by comparing, *W_mech_*, *S*_2*_M*_, at specific cycles number it was demonstrated that *S*_2*_M*_ has the same variation trend as *W_mech_*, which can be represented by a power law. This law of variation is the same as the one characterizing the stress of the S/N curve.

Further, by using a suitable model and a data calibration procedure, it was demonstrated as the stresses of the S/N curve can be obtained via the infrared signal acquisitions in a rapid way. In effect, the stresses obtained by using the damage state parameter *S*_2*_M*_ (*σ_Wtherm_*) lie in the 95% of the confidence interval and approximate the stresses of the S/N curves very well.

In view of the achieved outcomes of the present research, it is possible to highlight that the use of the present approach could allow:

- the realization of a real-time damage monitoring,
- the assessment of the fatigue behaviour of real components where it is difficult to measure the area under the hysteresis loop by means of an extensometer, and
- the assessment of parameters capable of validating the theoretical and numerical models.

It is clear that the present approach requires specific parameters calibration. In view of this, in this paper, the adopted parameter values are valid, specifically in the case of CFRP exhibiting a quasi-isotropic stacking sequence.

Further works will be focused on:

- the validation of the present approach on real components; and,
- the determination of S/N curve by using just thermal data.

## Figures and Tables

**Table 1:** Mechanical properties.

Property	Mean Value * [MPa]	Standard Deviation * [MPa]
Young’s modulus	55,800	1600
Ultimate Tensile Strength	825	85

* value over 5 tests.

**Table 2:** Loading table.

Cycles	Maximum Stress [MPa]	%UTS
1765	660.75	80
2875	660.75	80
6500	619.25	75 *
12,323	619.49	75
60,041	578.16	70 *
75,461	578.16	70
291,610	536.86	65 *
300,334	495.56	60 *
2,000,000	412.97	50 *
2,000,000	412.97	50

* indicates those tests that have been also monitored with the IR camera.

**Table 3:** Damage index parameter.

Stress [MPa]	*W_mech_* [10^6^ × J/m^3^]	*S*_2*_M*_ [Unit Signal]
412.50	0.50	6.46
495.75	0.65	8.12
577.50	0.74	10.32
536.25	0.68	9.10
618.25	0.85	12.08

**Table 4:** Stress obtained by using *W_mech_*, *W_therm_* as input for the Equation (9).

Cycles of the S/N Curve	$\sigma_{W_{mech}}\;[\mathrm{MPa}]$	$\sigma_{W_{therm}}\;[\mathrm{MPa}]$
2,000,000.00	447.97	481.17
300,334.00	504.92	526.28
60,000.00	596.91	580.58
290,000.00	547.14	551.13
6500.00	611.56	620.61

## Nomenclature

*E_s_*	energy stored in the material a cycle [J/m^3^]
*E_d_*	energy dissipated as heat in the material a cycle [J/m^3^]
*N*	actual number of cycles
*N/Nf*	ratio between actual cycles and cycles at failure
*Q*	energy exchanged between material and environment in a cycle [J/m^3^]
*UTS*	ultimate tensile strength of the material [MPa]
*R*	loading ratio
S	thermal signal
*S_2_m_*	processed value of the second harmonic amplitude of the thermal signal: mean value in the gage length
*S*_0_	mean temperature signal
*S*_1_	amplitude of the first harmonic amplitude of the thermal signal
*S*_2_	the second harmonic amplitude of the thermal signal
*S*_2_*M*_	mean value in the steady state condition of the S_2*_m*_ data series [Unit Signal]
*W*	total energy input per unit volume in a cycle [J/m^3^]
*W_mech_*	mean value in the steady state condition of the *W* data series [J/m^3^]
*W_therm_*	total energy input obtained by means of the calibration between *W_mech_* and *S*_2*_M*_ [J/m^3^]
Δ*U*	internal energy changes of the material a cycle [J/m^3^]
Δ*σ*	is the stress amplitude
*ε*	actual strain of the hysteresis loop
*σ*	actual stress of the hysteresis loop
*σ_Wmech_*	stresses obtained by using Ellyn’s model and *W* data series [MPa]
*σ_Wtherm_*	stresses obtained by using Ellyn’s model and *W_therm_* data series [MPa]
*φ_i_*	phase shifts of the harmonics (*i* = 1,2).
